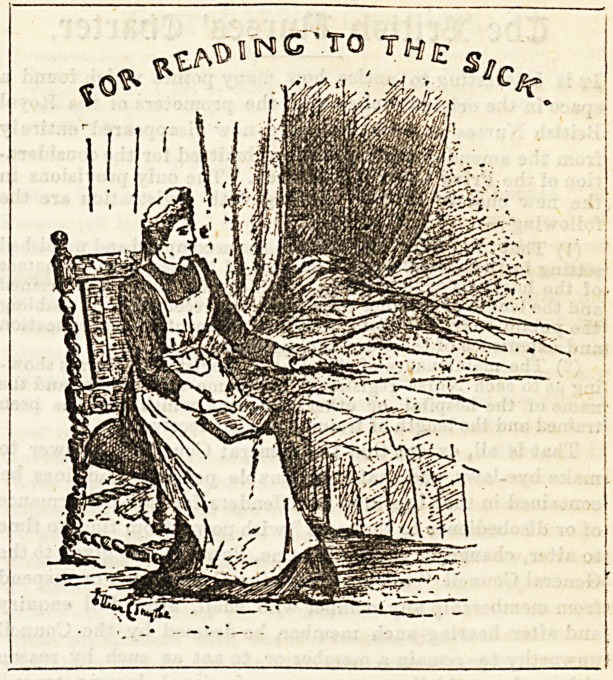# The Hospital Nursing Supplement

**Published:** 1892-03-26

**Authors:** 


### "The Hospital" Nursing Mirror.

Being the Extra Nursing Supplement of "The Hospital" Newspaper.

Contributions for this Supplement should be addressed to the Editor, The Hospital, 140, Strand, London, W.C., and should have the word "Nursing" plainly written in left-hand top corner of the envelope.

### En Passant.

**Figures for Nurses to Note and Remember.**—There is so much exaggeration and inaccuracy of statement about just now in regard to certain nursing matters that it may be well to give the actual facts. It has been claimed over and over again by speakers and writers in the interests of the Royal British Nurses' Association that the nurse members number 3,000. Again, in the first resolution passed at the meeting at the Mansion House on Friday (18th inst.) it was said that the Royal British Nurses' Association "includes one-fifth of the whole number of trained nurses estimated to be at work in this country," the member roll being given by the speakers as 3,000 nurses. We have examined the accounts published by this Association in their reports and journal for each of the periods for which they have been published, and we find, as a matter of fact, that taking the most favourable view the number of members, including doctors, matrons, sisters, and nurses, of the Royal British Nurses' Association for the year ending June 30th, 1891, was 2,043, and of nurse members alone 1,840, and no more. The following two tables give the figures :—

Actual Number of Paying Members of all Grades on the Books of the R.B.N.A. in the Periods Ending June 30th, 1889, 1890, 1891.

| Period or year ended June 30th. | Life Members. | | Doctors and Matrons at 10s. 6d. per annum each. | Nurses at 2s. 6d. per annum each. | Total Members. |
|---|---|---|---|---|---|
| | Matrons and Sisters. | Nurses. | | | |
| 1889* | 28 | 230 | 452 | 2,746 | 3,456 |
| 1890 | 3 | 111 | 170 | 1,687 | 1,971 |
| 1891 | 3 | 171 | 169 | 1,328 | 1,671 |

Total Members of R.B.N.A. under all Heads who Paid Subscriptions in Each Year, 1889, 1890, 1891.

| Period or year ended June 30th. | Life Members. | | Doctors and Matrons at 10s. 6d. per annum each. | Nurses at 2s. 6d. per annum each. | Total Members. |
|---|---|---|---|---|---|
| | Matrons and Sisters. | Nurses. | | | |
| 1889* | 28 | 230 | 452 | 2,746 | 3,456 |
| 1890 | 31 | 341 | 170 | 1,687 | 2,229 |
| 1891 | 34 | 512 | 169 | 1,328 | 2,043 |

*19 Months nearly.

It will be observed that the number of subscribing members, including all grades, has steadily fallen in each year. Thus, they have decreased from 3,456 for the 19 months ended June 30th, 1889, to 2,229 in the year ended June 30th, 1890, and to 2,043 for the year ended June 30th, 1891, the latest date for which the audited accounts are available. It is worthy of note that, whereas in the two first reports the actual number of members was given, as well as the receipts, in the last published statement, it is a significant fact that these numbers are omitted altogether. By a little careful calculation, we have ascertained that the life subscriptions for that year, which amouted to £195 6s., represent apparently three life subscribers of five guineas each and 175 life subscribers of one guinea each. The annual subscriptions, amounting to £254 14s. 6d., apparently represent 169 doctors and matrons at 10s. 6d. each, and 1,328 nurses at 2s. 6d. each. It thus becomes apparent that the actual numbers of nurses who at the present time can be properly regarded as members of the R. B. N. A. has fallen to 1,840, neither more nor less. These are precise figures taken from the audited accounts, and we shall be glad to have some explanation from the Council of the R. B. N. A. of the fact that the speakers at the Mansion House made so serious a mistake as to represent a members' roll, including really only 1,840 nurses, as one of 3,000 nurses. We have no doubt that this point will be thoroughly gone into before the Privy Council, but the sooner it is cleared up the better. It is, in any case, satisfactory to find that the nurses of this country have shown so much common-sense, as these figures prove them to possess, in reference to an association which has unfortunately been the means of producing more discord and ill-feeling than probably any organisation of the present century.

**The London Hospitals League.**—This excellent League, founded by the Lady Constance Howard, is in want of more members, and nurses might bring its merits to the notice of private patients. The subscription is three shillings a year, and each member has to undertake to make four garments annually for the good of the patients in the metropolitan hospitals. A list of suitable garments is given on the prospectus, and any further information can be obtained from the Hon. Sec., Lady Constance Howard, 34, Evelyn Gardens, Cranley Gardens, London, S.W.

**Work for Ladies.**—If any of our readers have time to spare they cannot do better than place it at the disposal of the Matron of the "Home and Hospital for Incurable Children," Maida Vale. These poor little folks are so terribly helpless that very few of them can even feed themselves, and it is at meal times that the assistance of one or two young ladies would be warmly welcomed. Help in amusing these afflicted children for an hour or two daily and in ministering to them in various ways, would also be of practical value, and would be much appreciated by those regular workers whose whole lives are spent in serving these little ones. Surely there are many ladies who can spare a few hours a week, and who will gladly give these hours freely and regularly to help these most helpless little mortals. To spend a morning in such an atmosphere of love and devotion as pervades this little institution, is to receive a lesson which might benefit each one of us!

**Short Items.**—Charles Matley, an old soldier who drove Miss Nightingale in the Crimea, and who always called himself "Florence Nightingale's coachman," died in Stepney Workhouse last week.—The Lady Superintendent and nurses of the Middlesboro' Nursing Association paid 5,590 visits last year. Funds are satisfactory.—Lucky Blackburn! Mr. and Mrs. Harrison have undertaken to furnish the new Nurses' Home; we congratulate the town on having such generous inhabitants.—The Lady Superintendent of the Jersey Institution was warmly thanked for her services at the late annual meeting.—Southport has three district nurses and a midwife. The expenditure last year was £203; this is economical.—We have been gratified by the receipt of a letter from the M. S. Hospital, Syria, where a lady medical missionary is working, who is a regular reader of ours.—The Naval Sisters a Stonehouse are very busy just now, as 100 boys from the training ships are in hospital from various causes.—Mrs. Denny, professional nurse, was summoned on Monday for assault, and bound over to keep the peace.

### On the Nursing of Children.

### VII.—THE SPREAD OF INFECTION.

This is a mattei which affects the whole community, and therefore it is impossible to dwell too seriously upon it.

We hear of a school closed because diphtheria, or measles, or scarlet fever has broken out amongst the pupils, and we read of similar trouble in workhouse or district schools, or even in hospitals, where wards have to be temporarily shut up for a similar reason.

In the opening speech at this year's Congress of Hygiene, the Prince of Wales was loudly applauded for his remark on the subject of preventable diseases. He said, "If preventable, why not prevented?" And if every woman took that lesson to heart, it would assuredly cause a great decrease in the number of those who fall victims to infectious diseases.

To begin with, we often hear the argument advanced that "someone" had said that such and such a precaution "was unnecessary."

Probably the "someone" knew little about the matter, and made the remark with no thought of its personal application. But it is well for us to believe that no precaution should be looked upon as needless, although diverse opinions exist as to the relative importance of preventive measures.

The woman who succeeds in keeping her school, her ward, or her home free from an epidemic has acquired, perhaps unconsciously, a vast amount of practical knowledge of hygiene and sanitation.

As everyone in charge of a group of children is liable to be confronted at any moment with a "rash," is is lucky when the system of supervision is so thorough that the presence of the "redness" is detected on its first appearance. In that case it is often possible to avoid similar trouble for the other children by isolating the first victim at once, and by removing everything belonging to him at the same time to the fresh room. Probably in 24 hours the doctor will be able to decide if a serious illness need be apprehended or if the rash is only one of those trivial eruptions to which children are liable.

It is a fatal mistake to wait until after a doctor has been summoned to take steps for isolation; there should be no time lost, and if the case proves serious this is of great importance; whereas if it chances to be only a temporary ailment there is no harm done by the precaution, and the quarantine can cease after a short period.

The half measures which used to be in vogue, such as "carbolic sheet" round the cot, are happily out of date, for it is difficult to imagine anything more absurd or ineffectual as a safeguard.

The other day a lady, possessed of plenty of common sense on most points, astonished a nurse very much by relating how very angry she was with the family doctor, adding, "and my husband and I have decided never to call him in again!" The explanation of her wrath being his behavour when summoned to one of her children. The boy was very feverish and red, and the visit was paid in the evening, and the doctor said, "I cannot decide to-night what is the matter, but we shall do wisely to treat him as if we were sure that he had scarlet fever." A most wise decision this seemed to the nurse, especially when she heard that the other children escaped the infection, but the lady will maintain to the end of her life that the doctor was an alarmist, and did not consider her feelings as he might have done, by waiting till the presence of scarlet fever became a certainty.

Of course, isolation, and nursing, too, are far more difficult in a private house, where the space is necessarily limited, than in an institution, where there are, or should be, spare rooms; also the fumigation afterwards is seldom so thoroughly done in the former, on account of the greater abundance of furniture and other valuables, which people dread spoiling. A habit of hoarding also tends to increase and to continue the dangers of infection, for hoarded-up books, toys, and articles of clothing, which perhaps are left in the open-air for a day, or submitted to a little perfunctory fumigation are frequently considered safe to put away! and "safe" they certainly are to secure further cases of illness when they are brought into use again.

Surely a little extravagance would be not only blameless, but commendable at such times as this; complete destruction by fire of all doubtful things, and a strong carbolic bath for those which can prudently be preserved.

A collection of old scrap-books caused more than one outbreak of illness, until it occurred to somebody to clear out the whole cupboard, and send off its contents with the last little victim who was dispatched to the Fever Hospital.

Another outbreak of fever might well be traced back to the unprincipled, or ignorant, maid, who took her mistress's new dinner dress into the sick-room, where her fellow-servant was in charge of a child desquamating after scarlet fever. If everybody were conscientious, how much trouble the world in general would escape.

In private nursing the prevention of the spread of infection lies to a great extent in the nurse's own power; she is trained to the observance of precautions, and she must remember that the members of the household are probably quite ignorant of the ways in which the danger exists. She must therefore never relax her vigilance from the moment when she is put in charge, to the time when every detail of the disinfection has been faithfully fulfilled, and her patient is judged safe to mix once more with his companions.

Several cases of measles occurring in a children's hospital, brought to light eventually a curious detail; the child who first showed the rash had only been in the ward for twenty-four hours, and it was afterwards discovered that there were other cases amongst the children who had been attending the same school as herself. This, however, did not explain the cause of three other children sickening rapidly, who had not been in immediate contact with her. But a wise old nurse cross-questioned the night nurse, who was rather new, and not quite satisfactory, and it transpired that, to save herself trouble, this woman had lazily washed the unfortunate children with the same piece of flannel as she had used for the new child. Of course, this unclean proceeding was contrary to all rules, but the mischief was done and a bitter lesson taught to the thoughtless and untrustworthy nurse.

If each fresh child admitted into a hospital, into a children's ward especially, be carefully examined by an experienced nurse before, as well as after, his bath, it is possible to keep out a great deal of danger, for many true stories can be told of the poor little creatures who are taken in for some accident or new ailment, and who are found to be "peeling"; or the mother, who has denied to the doctor the presence of anything "catching" in her home, often forgets caution when talking to nurse and owns to having "two others down with diphtheria," and "this one is just over it." And may even describe "one at home lying dead, and another terrible bad with measles."

She must not be hardly judged for the deceit and ignorance which she displays. She used the former to ensure getting one child off her over-worked hands, and the want of thought which led her to bring her child into the carefully guarded hospital ward had never struck her as culpable.

But no one who is responsible for the nursing of children should shrink from taking increasing trouble in obtaining the true history of each new case which comes into their hands. It needs much patience and infinite tact, and both should be devoted to this important detail.

If there be no unsanitary condition of the building itself, there should be no epidemics in a hospital; they are certainly "preventable" and they should be "prevented."

The visits of the patients' friends are a fruitful source of danger, and great care should therefore be exercised in their admission. Of course it is only natural and right that parents should come and see their children; but when there are other little ones ill at home, surely no momentary gratification justifies the mother's admission amongst a number of small patients already sick or suffering. She, poor soul, has to keep on the same dress and shawl in which she has ministered to her feverish baby, perhaps, and her first impulse is to kiss and embrace this other child without any knowledge of the risks he thus runs. Such proceedings should be guarded against as much as possible, and no little visitors should ever be allowed a place on the bed of the invalid in hospital.

*(To be continued.)*

### Fresh Fields.

THE place in which Her Majesty's Sisters have prospered most, the place in which we English suffer from ill-health most and most appreciate luxury, is India.

And yet in India there are few trained nurses for the vast population of railway officials, engineers, merchants, lawyers, and all classes of civilians.

This subject was brought forward in an article called "Wanted, Sick Nurses for India," by Mrs. Cuthell, which appeared in the *National Rev ew* for May last. It was repeated in an article on "The Anglo-Indians," by E. L. Robertson; it has been touched on by various writers, and is referred to in "In Tent and Bungalow," a book just published by Methuen and Co. But it has surely never been brought fairly before nurses, or this new field on which fortunes can be earned had before this been occupied.

Could not a group of thoroughly trained nurses, competent women with common sense, able to appreciate that "nothing venture" ever means "nothing have," form themselves into a co-operation, and go over to India to start the first private nursing institution there? They would need to have each £100 capital for a start. They had better have a committee, to draw up plans and rules for their guidance, and they had better elect one of their number as their Lady Superintendent. This would be just the time to thrash out this proposal, for not only is Mrs. Cuthell in this country (Oaklawn, Wootton Isle of Wight), but Mrs. Sheppard, known for having organised the Poona Hospital, is also in England, though we do not know her address. There ought to be no difficulty in getting together a committee of medical men and ladies who have a knowledge of Indian life, and also no difficulty in getting an Anglo-Indian nurse to take the responsible post of head of the adventurous little band.

The first step for anyone to take who is interested in this scheme, is to read Mrs. Cuthell's article in the *National Review*, which, thanks to free libraries, anyone can do by dint of a little time and trouble. Other books on India, and conversation with Anglo-Indians, would be helpful. It is no light thing to take up fresh work in a far country; it is only suitable for strong, well-trained women in the prime of life who add to their love of nursing a spirit of adventure, and who can cheerfully risk their little capital in the hope of success in the new sphere. But supposing the little band of pioneers to consist of picked women, cheerful and capable in the face of all difficulties, and ready to rough it if necessary, they would surely not only win a comparative fortune for themselves, but also beat a path along which their less adventurous sisters could tread to competency.

It would be most necessary that any nurse going to India should hold the L.O.S. diploma and a certificate of general training; there are plenty of so-called monthly nurses out there who are, however, untrained, and not ladies by birth. In India the native servants nearly all eat and sleep out of the house, and there are no quarters for any save the family and visitors, so that the lady-nurse could adapt herself well to the system of living.

### "THE VALLEY OF THE SHADOW."

WE read in the Bible that "All things come alike to all: there is one event to the righteous and to the wicked," and in our own experience we see that high and low, rich and poor, good and bad, man and beast, alike go sooner or later to their long home. Many of us have lost relatives and friends who are still very dear to our hearts, and have mourned for them as those who can never return to us again, while we fail to remember that we shall go to them.

Ah, dear friends, we should sometimes dwell on the thought of our departure from this life, and though the sickness we are now suffering from may not be unto death, by God's mercy we may have another span of life given us in which and by which we may glorify Him—yet it is uncertain. He knoweth best—the wise Physician will raise us from the bed of languishing, or take our souls into His own keeping.

If we are true Christian believers to us there is no death, only a falling asleep, to awake in the presence of our beloved Lord, and we will lie still and pray, knowing that His angels stand round about us. We will ask Him to keep His promise to be with us through the dark valley of the shadow, and that His rod and staff will comfort us when we reach the river's brink. There are some who, unhappily, have not made their peace with God. If we are of such let us arise quickly and cast ourselves at the feet of Jesus. We will make a full confession of our sins to Him with tears of penitence and with humble faith in His merits alone wait for forgiveness. Those who come to Him He will in no wise cast out. He proclaims to us as He did to the mourning sisters of Bethany, "I am the Resurrection and the life, he that believeth in Me though He were dead—dead even in trespasses and sins—yet shall he live!" And "Whosoever believeth in Me shall never die." With Christ for our champion death has lost its sting, the grave is no longer the victor. May it be our happy lot to fall—

Asleep in Jesus! Blessed sleep!
From which none ever wake to weep!
A calm and undisturbed repose,
Unbroken by the last of foes.

Asleep in Jesus! oh, how sweet
To be for such a slumber meet!
With holy confidence to sing
That death hath lost its venomed sting.

### The British Nurses' Charter.

It is interesting to notice how many points which found a space in the original scheme of the promoters of the Royal British Nurses' Association, have now disappeared entirely from the amended draft charter submitted for the consideration of the Privy Council last week. The only provisions in the new charter directly dealing with registration are the following:—

(1) That . . . . a List of Nurses has been compiled and published setting forth the names and addresses of Nurses, with the names of the hospitals or institutions at which they have been trained and the length of training which each has received, thus enabling the public to form a more accurate judgment of the education and experience of the Nurses so registered.

(2) The maintenance of the List or Register of Nurses showing as to each Nurse registered, her name and address and the name of the hospital or other places at which she has been trained and the length of training she has received.

That is all, except that the General Council has power to make bye-laws, and that "reasonable penalties and fines be contained in such bye-laws on offenders for non-performance of or disobedience to the same" with power from time to time to alter, change, or annul the same. Power is also given to the General Council "to expel from the Corporation or suspend from membership any member who shall, after full enquiry and after hearing such member, be deemed by the Council unworthy to remain a member or to act as such by reason either of moraldelinquency or professional incompetence" under certain conditions and restrictions as set forth in the charter.

These powers seem to be surprisingly modest, and ineffective if we may judge them by the clauses which were inserted in the original draft charter which was submitted to a meeting of the Council on July 12th, 1889. They ran as follows:—

Registration Board.

The General Council shall appoint a Registration Board for nurses and midwives.

The said Board shall consist of (1) duly qualified medical practitioners, including obstetric physicians; (2) professional nurses; and (3) midwives; in such numbers respectively as may be prescribed by the bye-laws for the time being in force.

The said Board shall form, control, publish, and carry on (1) a register of nurses, male and female, to be called "The Nurses' Register"; and (2) a register of midwives, to be called "The Midwives' Register."

The said Board shall determine from time to time what tests shall be satisfied by candidates for registration, as evidence that they are possessed of the necessary character, skill, and knowledge, for the efficient tending respectively of the sick and of women in labour.

The said Board shall have full power at any time to temporarily or permanently remove from the registers the name of any nurse or midwife who shall appear to the Board or to the majority of two-thirds thereof, unworthy by reason of any defect of moral character, professional competence or trustworthiness, to remain further upon the registers, or either of them, provided always that no name shall be removed from the registers until the accused nurse or midwife has had the opportunity of appearing personally, or by proxy, before the Board, to show cause why such suspension or total removal of her name from the register should not be effected.

Fees for Registration.

The Registration Board may charge such fees for the Registration of Nurses and Midwives as may be prescribed by the Bye-laws of the Association for the time being in force.

The Registration Board may, in their discretion, register, without examination or other test of professional skill or knowledge, the name of any person who at the date of these presents shall have been engaged for three years or more in practice as nurse or midwife for payment, provided that no person shall be so registered without examination or test, except within six months from the date of these presents.

The Registered Nurse.

Any person whose name shall have been entered by the said Board on "The Register of Nurses," may use the title "Registered Nurse;" and any person whose name shall have been entered by the said Board on "The Register of Midwives," may use the title of "Registered Midwife," provided that the right to use such titles, or either of them, shall cease if and when the name of the person using them shall for any cause have been removed from the Register.

No doubt the promoters have exercised a wise discretion in leaving the midwives severely alone so far as they are concerned. It will be interesting to hear why the Registration Board has been given up, together with the title of "Registered Nurse," and to hear what value any nurse can properly attach to having her name entered in a register which will compensate her for the fee charged, and which requires no test as to the possession of the "necessary character, skill, and knowledge," and provides in no way for the temporary or permanent removal of the unworthy for any "defects of moral character or professional competence or trustworthiness." We commend in this connection a very pertinent leading article on this subject which appeared in the *British Medical Journal* of the 19th inst., which every nurse should read and ponder over as, indeed, should all who are interested in the question or have to deal with it editorially or judicially. There is no doubt, as the *British Medical Journal* forcibly states, that "no one would ever think of embodying such a scheme as that contained in the charter submitted to the Privy Council in a Bill before Parliament." The *British Medical Journal* also adds, with truth and force, that the charter is "backed by high recommendations given, we apprehend, somewhat carelessly." It is essential, therefore, that Sir Richard Quain and Sir Dyce Duckworth should answer the four pertinent questions put by the *British Medical Journal* to these eminent medical men, the categorical answers to which are expected by the profession as well as by the managers of the institutions, and the examiners who have made nursing what it is in this country to-day.

Finally, it will be seen by the extracts we have given from the present and the former charter that for all practical purposes the registration of nurses, in any proper meaning of that term, has been virtually abandoned by the Royal British Nurses' Association, as well as the registration of midwives. Assuming that a Royal Charter as at present drawn, were granted to-morrow, no intelligent trained nurse of high character would be foolish enough to pay half a guinea, and so hamper herself by being associated with anybody who might be admitted to such a list as that which the Royal British Nurses' Association now seek power to set up. What is now proposed is in effect an ordinary directory, and not a register at all, as that term is understood in relation to the registration of doctors, dentists, plumbers, and others.

### Round an Asylum.

There is generally one distinct characteristic to every institution; some asylums are "smart," the attendants all stand at attention when addressed, the wards are spotless, the patients all seated in rows along the walls. Other asylums are marked by extraordinary freedom, or by the high tone of the attendants, or by the clever construction of the building. On leaving Colney Hatch, after a wander round the wards, the most distinct impression left on the mind is that it is a homely asylum, there is a pleasant ease, an atmosphere of kindliness, which distinctly impress the casual visitor. Is it the remembrance of one of the medical officers, with his back to the fire, smiling down on the group of patients gathered round him, and pestering him with questions? Is it the remembrance of the crimson curtains, or the big white cat curled up on a patient's lap? Probably it has its rise in these and many other half-perceived details; anyway the impression is distinct that there is a homelike atmosphere about the wards of Colney Hatch.

As for statistics, they must remain in reports; there are about 13,000 patients on the female side, and over 100 nurses to attend to them, about 30 being charge nurses. But exact figures are a burden to the memory. The nurses are on duty about thirteen hours daily; they get three and a-half days off in the month. Most of the charge nurses have been for years in the service of the institution, and the Matron has been there eighteen years. The nurses are said to have one great fault, that they do not save for the proverbial rainy day. They ought to have joined the Pension Fund and gone to Marlborough House last June with the attendants from Northampton. The uniform is neat and distinctive—a blue frock with red collar and red waistband; the number of the nurse's ward being affixed to her band. Out of doors a very becoming blue bonnet and little red shawl are worn. There are courses of lectures for the attendants. The patients are, of course, paupers; but in every ward there seemed to be some-one capable of taking advantage of the pianos provided; one of the saddest sights was a Scotch girl playing her native airs in a jerky manner, stopping in the middle of "Annie Laurie" to pass on to "Rothsay Bay," and finally breaking a note over the final bars of "Bonnie Dundee." "Ou, aye, doctor," was her expression of consent, and when she asked, "Is that your hāt?" the accent recalled many pleasant days on the Scotch shores. Another sad case was that of a young German girl, with fresh pink cheeks and blue Teutonic eyes. She could not speak a word of English, and no one present could speak German. She watched the faces of the visitors so keenly, evidently trying to guess about what they spoke. In the infirmary ward was a baby—a poor little wretch which had entered upon life in the midst of madness and imbecility, and whose future it were hard to be hopeful about. The refractory wards at Colney Hatch are wonderfully full of ornaments and free from signs of restraint. True, all doors are locked all over the building, and porters keep guard over the entrance. There is small chance to escape, but there is every chance to break (either windows or china) if the patients are so disposed. As a matter of fact, they are not very destructive, save in the matter of clothes; one old woman was dressed in a stout quilted cotton garment that no human fingers could tear.

There is a very bright convalescent cottage in the grounds that the patients are passed on to as they get better, and the whole system seems satisfactory.

### Presentation.

Our readers will remember that we referred some time since to the testimonial being raised for Miss Minet who, for 20 years, has worked in connection with the Stratford Nursing Home and Children's Hospital. We are glad to say that this testimonial has reached £1,000, and has been invested in Miss Minet's behalf in the names of two trustees. Four hundred and four persons subscribed, the subscriptions ranging from 1d. to £100. In acknowledging the gift to Mr. Mason, Miss Minet says: "I know not how I can better convey my deeply grateful thanks to the committee and to all those many kind friends who have so generously combined to present me with a gift of £1,000—a gift as unlooked for as it was uncalled for—than by placing myself in your hands, who have acted as Secretary, and begging you to assure all the numerous subscribers of my most sincere appreciation of their great kindness. I feel too deeply to say all I would wish to say. Words quite fail me, and I can only trust that the generous donors will understand how impossible it is for me to thank them as I would fain do."

### Wants and Workers.

*To Cottage Hospital Matrons.*—A partially-paralysed girl, neat and clean, and capable of scrubbing and sweeping, needs a home in return for her board and lodging. Particulars from Miss Glossop, 3, The Paragon, Richmond, Surrey.

### Everybody's Opinion.

*[Correspondence on all subjects is invited, but we cannot in any way be responsible for the opinions expressed by our correspondents. No communications can be entertained if the name and address of the correspondent is not given, or unless one side of the paper only be written on.]*

### A PERSECUTED HOSPITAL.

Mrs. Robert Hunter writes: In an article headed "A Persecuted Hospital" in your issue of the 12th, you speak of ladies formerly connected with the London and other hospitals, who some years since were offended by a lady official of the London Hospital, and you attribute the "attack" lately made on the nursing department of tha hospital to the consequent implacable hatred since borne this official by the lady or ladies in question. As I am the only lady whose name has frequently been mentioned by you in your reports of the proceedings at the quarterly Courts of Governors of the London Hospital as taking an active part in those proceedings, your article is calculated to create the impression that I am one of the ladies referred to as actuated by personal motives in the course I have taken. This impression must be strengthened by an allusion to my name in an article entitled "Exeunt" on the page opposite to article above referred to. I must therefore ask you to give equal prominence with the Persecuted Hospital article to a disclaimer on your part in your next issue of any intention to refer to me in that article, and to my statement that I have never at any time had any personal acquaintance with any lady official (unless nurses are so called) of the London Hospital; that consequently I have never been directly or indirectly offended by any such official, and that I have never at any time had any connection with the London or any other hospital except as a subscriber.

[We were in no way referring to Mrs. Hunter in the article quoted above; those who are acquainted with nursing and hospital affairs, as most of our readers are, must have known perfectly well to whom we referred.—Editor.]

### Appointment.

Miss Mary E. Arnold has been appointed Parish Nurse at Slough. Miss Arnold trained at King's College Hospital, and was afterwards at the London Association of Nurses, New Bond Street. She commenced her new duties on March 1st.

### Notes and Queries.

### Queries.

*Massage and Electricity.*—Which is the official school of massage in London? Do Messrs. Coxeter's Dry Cell Batteries last a reasonable length of time, and about how long is that time?—*American.*

### Answers.

*Mrs. Jackson.*—Five shillings and sixpence an hour is the usual fee for a certificated masseuse, but some skilled masseuses command as much as a guinea an hour.

*Exeter.*—It is not usual for a nurse to make any extra charge for performing the last offices for the dead.

*Enquirer.*—The steamers start from Hull; the fares vary: a fortnight's trip would probably cost you from £25 to £45. Cheap trips are arranged yearly by the Polytechnic, by Messrs. Cook, and by Gaze and Sons. Any of these would give you full particulars. The Polytechnic trips are very cheap.

*M. L. H.*—We know of no fund which would help you; the Benevolent Branch of the Pension Fund would lend you the money if you are a member, or possibly the Midwives' Institute, 12, Buckingham Street, Strand, might help you.

*Worcester Institution.*—Through a printer's error, we lately stated that the cases visited by the district nurses of this institution were 425, instead of 3,425.

*P. M. H.*—If the boy is a Roman Catholic, apply to the Hospital of St. John and St. Elizabeth, Great Ormond Street, W.C., or apply to the British Home for Incurables.

*Catherine L. M. E.*—Get "The Hospital Annual," price 3s. 6d., from this office; we cannot write out all particulars, or write to the Matrons of the Evelina and other children's hospitals.

*Laura L.*—Write to the Matron of the hospital mentioned in "Amongst the Lepers" in our issue for March 5th.

*To Correspondents.*—Please address all news to the Editor at this office, not to anyone personally.—*H.M.*

### Off Duty

### A Baker Street Mystery.

"I think Baker Street Station is the nastiest and grimiest in London!" This expression of public opinion emanated somewhere from out a crowd of individuals who were struggling along the platform towards the stairway notified by a big white lettered board to be the "way out." Two clerkly looking youths, who were of the multitude, heard it, and exclaimed, as if in one unanimous breath, "Hear, hear!" The darker complexioned of these two young men—he with the thin face and aquiline nose—suddenly missed his companion from his side. "Where are you off?" he cried to the retreating figure, but received no response; so he stood still until the said figure advanced again upon him. Then he reproached him amicably: "What a chap you are for newspapers, Jerrold!"

Jerrold, holding up the evening paper he had just purchased, replied grimly: "The Desterville Murder Case! Supposed Clue!"

The said Jerrold was baby-faced and fair, but his small eyes were very bright and interesting. "Wheugh!" he made utterance, when he and his friend had reached the stair-head, and were breathing the fresher air of the Marylebone Road. However, he seemed greatly interested in the murder case, for he kept on referring to it. "It seems odd that husband and wife must needs kill each other. There can be no doubt that this fellow Desterville cut his wife's throat, and just got away in time. The thing is to catch him now. He ought to be burnt slowly—"

"And then Madame Tussaud would glorify him for ever with a wax image in this place!" broke in the sharp-nosed youth, pointing to the rectangular pile of red brick and skylights which they were just passing.

"Confound it, yes. Eternal fame lies in that immortal Chamber of Horrors. You laugh, Phil! Ugh! it's chilly this evening—let's hurry home."

Soon the two friends reached a street narrowed to the view by two lofty, opposing walls of houses. Into one of these houses they gained entrance by a latch key, and then trudging up four flights of stairs reached their sitting apartment. It was a little back room shabbily furnished, but cosy in a way, with a double-bricked fire burning all it could (which was not much) in the grate, and a kettle simmering upon it. Their tea was laid out for them on the table where a lighted oil lamp shed its rays over tea cups, and bread and butter, and a half-used tin of sardines.

"I say, make the tea Phil, will you? while I see what's the latest about the murder," Jerrold asked and proceeded to spread his *Evening News and Post* under the lamplight, and then to read:—

"The Police are actively following up the new clue. A man answering to the published description of Desterville was seen in Baker-street yesterday. It is believed that he has not yet left the country, and precautions are being taken to prevent him from doing so. Furthermore, intelligence has come to hand which leads to the suspicion that the late Clara Desterville was not the only victim of this homicidal maniac."

"That's strong language!" Phil called out from the recesses of a cupboard where he was reaching for the tea-caddy. Jerrold drew his chair near to the fire-place, and stooped down to take off his boots. "Not a bit too strong," he said. "The man's a murderer, and nobody but a maniac could have cut his wife up the way he did. Ugh! the room feels very chilly to-night."

"It's going to freeze, I think."

"Baker Street, by Jove!" Jerrold mused aloud, throwing his boots into a corner, and pushing his feet into slippers. "That's uncomfortably close, eh Phil? He may be roaming about these parts this minute, for all we know."

"On his way to Madame Tussauds?"

"You're the most unimaginative chap, Phil—always talking nonsense. You know the new lodger: he's small, clean-shaved, and pleasant-countenanced—that's just Desterville's description."

"Oh, confound the murder!" Phil exclaimed. "Let me get at the kettle and warm the tea-pot. It's murdering cold, I know that. Make some toast, will you?"

Jerrold cut two huge rounds of bread, and, sticking a knife into the middle of each, stooped over the fire. "Look here, Phil!" he burst out presently. "Suppose you meet that man on the stairs when you are going to bed to-night!"

"I'll say good night to him."

"But think of the feeling in that dark corner, for instance. He could whip out a knife and throat you before you'd have time to look round, and everybody gone to bed—just think!"

"I do think very much that this room is uncommonly draughty to-night, somehow."

"See if the window is open, the blind is swinging about."

Phil moved to the window, and drew aside the blind. Suddenly the striped canvas fell out of his hand. "Good God!" he exclaimed, "lo—look!" He made a sign towards the window. Jerrold jumping up with the knives and toast in his left hand, pulled at the blind cord with his right. Outside was the back of another big block of buildings. Sprinkled all over this were windows. Some showed lights within drawn blinds, others were in darkness with or without drawn blinds; but there was one window which had a light within, and yet the blind—a Venetian one—remained open. Through this it was quite possible to see distinctly what was going on inside the room. A middle-aged man was carefully combing out a woman's long hair. Her head lay in his lap—but there was no body to it!

Jerrold, full of uneasy imaginations, murmured, "Small, clean shaved, and pleasant countenanced—that's him, Phil—that's Desterville, I'll bet a pound; and the woman is another victim. Ugh! what a cold-blooded beast it is to go on combing out the woman's hair he's murdered." Jerrold's grammar was not select at this moment. "I'm going to pull down the blind," he went on; "I've had enough of that sight. Besides, if the fellow sees us he'll bolt. Let him go on while we find the police."

It took not many minutes for them to find a policeman. Their discovery was considered very important, and other policemen were requisitioned, and intelligence was dispatched to the police-station. And soon the dwelling of the presumed murderer was surrounded on all sides by a posse of constables. Then a couple of detectives and Phil and Jerrold knocked at the door and asked for the gentleman who occupied the third story back room. "When did he come?" one of the detectives asked the servant.

"But yesterday, sir."

The detectives looked at one another significantly. They were surprised to find the man's room-door unlocked, but were fairly taken aback when, having entered, he just turned his head round to ask them what they wanted, and then went on calmly with his work, gathering up the long threads of hair dexterously in his comb. As no one answered him for a minute, he turned his head round again. At first his expression was amazement, then indignation; then a sudden light flashed into his eyes, and when the detectives and Phil and Jerrold were all nerving themselves ready to spring upon him, he burst into roar after roar of laughter until the very room shook, and the head in his lap rolled off down his legs to his feet, where a strong ray of light happened to fall upon it.

It was the head of one of Madame Tussaud's wax works unscrewed, and taken home by the man to be cleaned.

The murderer Desterville still remains at large.

## Figures and Tables

**Figure f1:**